# Isolation, Identification, Genomic Diversity, and Antimicrobial Resistance Analysis of *Streptococcus suis* in Hubei Province of China from 2021 to 2023

**DOI:** 10.3390/microorganisms12050917

**Published:** 2024-04-30

**Authors:** Yingjun Xia, Zhaoyang Wang, Yanli Hu, Pengfei Zhao, Jianhai Li, Li Zhang, Rui Fang, Junlong Zhao

**Affiliations:** 1 State Key Laboratory of Agricultural Microbiology, College of Veterinary Medicine, Huazhong Agricultural University, Wuhan 430070, China; 2018302120125@webmail.hzau.edu.cn (Y.X.); huyanli@webmail.hzau.edu.cn (Y.H.); pengfeizhao0513@163.com (P.Z.); ijiahai@webmail.hzau.edu.cn (J.L.); zhangli951010@163.com (L.Z.); fangrui19810705@163.com (R.F.); 2 Key Laboratory of Preventive Veterinary Medicine in Hubei Province, Wuhan 430070, China; 13615116152@163.com; 3 Key Laboratory of Animal Epidemical Disease and Infectious Zoonoses, Ministry of Agriculture, Huazhong Agricultural University, Wuhan 430070, China

**Keywords:** *Streptococcus suis*, MLST, pan-genome, antimicrobial resistance, ARG

## Abstract

*Streptococcus suis* (*S. suis*) is a zoonotic pathogen capable of causing severe diseases in humans and pigs, including meningitis, sepsis, polyserositis, arthritis, and endocarditis. This study aimed to investigate the biological characteristics of 19 strains of *S. suis* isolated from diseased pigs in Hubei Province between 2021 and 2023. Through bioinformatics analysis, we investigated the serotype, MLST, pan-genome characteristics, SNP, AMR, and ICE of the 19 *S. suis* isolates. Among the 19 *S. suis* strains, ten serotypes were identified, and serotype 9 was the most prevalent (21.05%). Ten new alleles and nine new sequence types (STs) were discovered, with ST28 and ST243 emerging as the predominant STs. The results of the pan-genomic analysis of *S. suis* indicate that there are 943 core genes, 2259 shell genes, and 5663 cloud genes. Through SNP evolutionary analysis, we identified a strong genetic similarity between SS31 and the reference genome P1/7. The analysis of antibiotic resistance genes revealed widespread presence of *erm*(B) and *tet*(O) genes among 19 strains of *S. suis*. This association may be linked to the high resistance of *S. suis* to lincosamides, macrolides, and tetracyclines. Integrative and conjugative elements (ICEs) and integrative and mobilizable elements (IMEs) were identified in 16 strains, with a carriage rate of 84.21%, and resistance genes were identified within the ICE/IME elements of 8 strains. Antimicrobial susceptibility testing revealed that all strains showed sensitivity to vancomycin and lincomycin but resistance to tilmicosin, tiamulin, amoxicillin, and doxycycline. This study contributes to our understanding of the genomic diversity of *S. suis* in Hubei Province of China, providing essential data for the comprehensive prevention and control of *S. suis* infections in China.

## 1. Introduction

*S. suis*, a capsulated Gram-positive coccus, has developed antibiotic resistance due to the abuse of antibiotics, causing significant economic losses worldwide. Not only does it infect pigs, but it also affects humans, leading to diseases such as pneumonia and meningitis [1]. The capsule, a critical virulence factor, differentiates *S. suis* into 35 serotypes (1–34 and 1/2) based on capsular polysaccharide differences, showing marked variability in pathogenicity among strains [2]. However, new research indicates that serotypes 20, 22, 26, 32, 33, and 34 do not belong to *S. suis* [3,4,5]. Serotypes 1, 2, 7, 9, 14, and 22 are predominantly pathogenic, with serotypes 2 and 14 linked to human diseases. Seroagglutination tests, considered the gold standard for serotyping, have been supplemented by polymerase chain reaction (PCR) methods targeting genes associated with capsular polysaccharide (CPS) production over time. However, PCR cannot differentiate between serotypes 2 and 1/2 or between 1 and 14 [6]. Due to single-nucleotide polymorphisms in the cpsK gene, whole-genome sequencing (WGS) remains the optimal method for distinguishing these serotypes [7]. WGS clarifies the bacterium’s epidemiology, origins, and evolution, assesses isolate relatedness, and identifies genetic features defining isolate subsets. Some studies suggest that a single genome does not fully reflect how genetic variation in bacterial species drives pathogenic mechanisms, whereas pan-genomic studies can better elucidate the genetic mechanisms behind phenotypic diversity and analyze species genome dynamics [8]. Guo et al. constructed a pan-genome from 19 *S. suis* strains, revealing that it comprises 1239 core genes and 2436 accessory genes, with 53 and 58 genes uniquely present in highly- and less-virulent strains, respectively [9]. Andrea Gori et al. conducted a pan-genome-wide association study on 1988 *Streptococcus agalactiae* strains isolated from different hosts and countries, identifying 279 specific genes related to virulence, disease, metabolism, and cellular mechanism regulation, including unique genes for pili, iron uptake systems, and quorum sensing in more invasive strains [10].

The abuse of antibiotics has significantly worsened antimicrobial resistance (AMR) in *S. suis*, emerging as a global concern in recent years. The rise in AMR cases has been noted in pigs and humans across Asia, Europe, North America, and Africa. Exceptionally high resistance rates have been observed among the tested antimicrobials for lincosamides, macrolides, and tetracyclines [11]. Although the resistance mechanisms of *S. suis* are not fully understood, previous studies have reported resistance genes and their products mediating resistance to specific antibiotics. Mobile genetic elements such as integrative and conjugative elements (ICE), plasmids, and insertion sequences play a critical role in the horizontal transfer of AMR-related genes. AMR genes carried by ICE is an important vector for natural transfer between different bacterial pathogens, including *S. suis* [12]. Therefore, monitoring the AMR spectrum and genotyping *S. suis* is essential for optimizing effective antimicrobial treatments and tracking the emergence of bacterial resistance. To deepen the understanding of the genomic and epidemiological characteristics of *S. suis* in diseased pigs, we collected samples from diseased pigs in Hubei province and isolated 19 *S. suis* strains. Subsequently, we conducted MLST typing, pan-genomic analysis, antibiotic resistance studies, and research on ICE elements for the isolated strains, providing valuable insights for the control and prevention of *S. suis*.

## 2. Materials and Methods

### 2.1. Isolation and Culture of S. suis

Over two years, from 2021 to April 2023, lung samples were collected from clinically diseased pigs across nine regions in Hubei Province. The samples were promptly transported to the laboratory under refrigeration. Deep tissue samples were aseptically excised using sterile scissors and then inoculated onto TSA plates supplemented with 5% fetal bovine serum. Following inoculation, the plates were streaked for isolation and subsequently incubated at 37 °C for 24 h.

### 2.2. Identification of S. suis Isolates

The 16S rRNA gene of suspected isolates was amplified by universal primers (27F:5′-AGAGTTTGATCCTGGCTCAG-3′, 1492R:5′-CTACGGCTACCTTGTTACGA-3′) [13] and *rec*N gene (F2:5′-CTACAAACAGCTCTCTTCT-3′, R2:5′-ACAACAGCCAATTCATGGCGTGATT-3′) [14]. PCR reactions were carried out using the following cycle parameters: initial denaturation at 94 °C for 10 min, followed by 35 cycles of denaturation at 94 °C for 30 s, annealing at 55 °C for 30 s, and elongation at 72 °C for 5 min. The PCR products were sequenced and analyzed, and gene sequences obtained were compared in the National Center for Biotechnology Information (NCBI) for homology using BLAST.

### 2.3. Extraction and Sequencing of the S. suis Genome

Bacterial genome extraction was carried out using the TIANamp Bacteria DNA Kit (Tiangen-Biotech, Beijing, China) according to the manufacturer’s instructions. All *S. suis* strains were sequenced using short-read Illumina (Illumina Inc., San Diego, CA, USA).

### 2.4. Genome Assembly and Annotation

The assembly of Illumina reads was performed using SPAdes (v1.1.0) [15]. The 19 genomes of *S. suis* were annotated using the Prokaryotic Genome Annotation System (Prokka) [16] and further refined using eggNOG-mapper v2 (http://eggnog-mapper.embl.de/, accessed on 10 February 2024) [17].

### 2.5. Pan-Genome Analysis of S. suis Isolates

Roary v3.11.2 [18] were used to perform pan-genome analyses by the GFF files generated by Prokka [16]. Accordingly, we obtained four different classes of genes belonging to ‘core’ (99% ≤ strains ≤ 100%), ‘soft core’ (95% ≤ strains < 99%), ‘shell’ (15% ≤ strains < 95%), and ‘cloud’ (0% ≤ strains <15%) groups, respectively. 

### 2.6. SNP Analysis of S. suis Isolates

The core genomes of *S. suis* strains were aligned using Snippy v1.5.3, calling the single-nucleotide polymorphisms (SNPs) and determining the core genome phylogeny [19]. The produced phylogenetic tree was visualized using R version 4.3.3 (ggtree version 3.10 package) [20].

### 2.7. MLST Analysis of S. suis Isolates

Seven house-keeping genes in the *S. suis* genome described previously were used to determine the STs of *S. suis* strains [21]. The *S. suis* multilocus sequence type (MLST) was retrieved from genome data using PubMLST (https://pubmlst.org/, accessed on 1 February 2024), and the untyped *S. suis* genome was uploaded to PubMLST to obtain ST information.

### 2.8. Antimicrobial Susceptibility Testing

The broth microdilution method determined the minimum inhibitory concentrations (MICs) of 12 antimicrobial agents from 10 classes against *S. suis*. The antimicrobials evaluated in this study included β-lactam antibiotics (amoxicillin), glycopeptide antibiotics (vancomycin), oxazolidinones (linezolid), phenicols (florfenicol), fluoroquinolones (enrofloxacin and ofloxacin), macrolides (tylosin and tilmicosin), lincosamides (lincomycin), pleuromutilins (tiamulin), aminoglycosides (streptomycin), and tetracyclines (doxycycline). *Streptococcus pneumoniae* ATCC 49619 served as the positive control. The resistance/susceptibility to each antibiotic was determined based on breakpoints established in CLSI (VET08-ED4 and M100-ED29) [22,23], and when not available, the literature was used [24,25,26].

### 2.9. Identification of Antibiotic Resistance Genes and ICEs

The resistance genes and distribution of Integrative Conjugative Elements (ICEs) and Integrative Mobilizable Elements (IMEs) in 19 bacterial strains were identified based on their whole-genome sequences using the online tools ResFinder [27] and ICEberg2.0, as reported by Liu et al. [28].

## 3. Results

### 3.1. Isolation and Identification of S. suis

**Figure 1:**
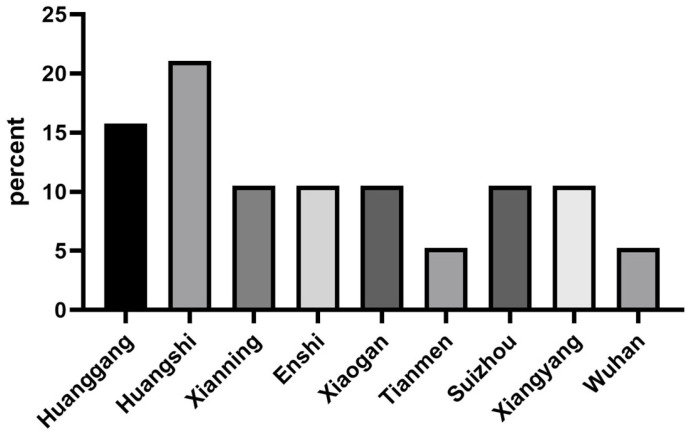
Proportion of *S. suis* isolates in each region.

Nineteen strains of *S. suis* were isolated from clinical samples submitted from Hubei Province, China, between 2021 and 2023. As shown in Figure 1, these samples were collected from different regions within the province, including Huangshi (4/19, 21.05%), Huanggang (3/19, 15.79%), Xianning (2/19, 10.53%), Enshi (2/19, 10.53%), Xiaogan (2/19, 10.53%), Tianmen (1/19, 5.26%), Suizhou (2/19, 10.53%), Xiangyang (2/19, 10.53%), and Wuhan (1/19, 10.53%). The tissue sources for these *S. suis* strains were the lungs (15/19, 78.95%), pericardial effusion (2/19, 10.53%), lymph nodes, spleen (1/19, 5.63%), joint effusion, and pericardial effusion (1/19, 5.63%). Detailed sample information is presented in Table S1.

### 3.2. Typing of S. suis Isolates

**Figure 2:**
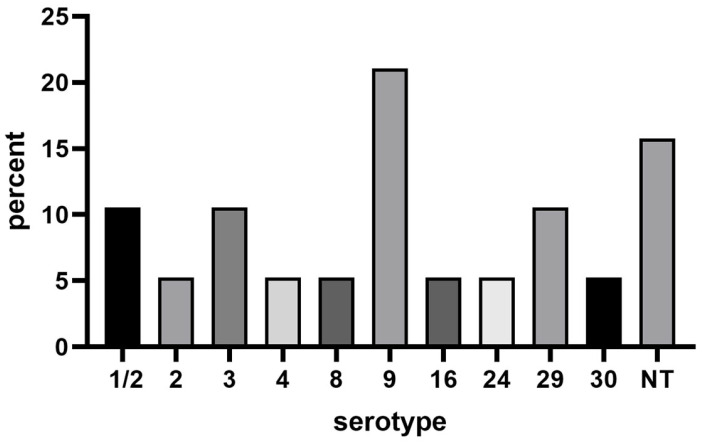
The prevalence of serotypes of *S. suis* isolates from Hubei in 2021–2023. NT = Nontypeable isolates.

The results of the serotyping of 19 isolates from Hubei during 2021–2023 are shown in Figure 2. This study identified the serotypes of 19 strains of *S. suis*. A total of 10 different serotypes were identified, with the predominant serotype being serotype 9 (4/19, 21.05%). The serotypes included two isolates of 1/2, 3, and 29, one isolate of 2, 4, 8, 16, 24, and 30, and three isolates that remained unidentified.

### 3.3. Multilocus Sequence Typing (MLST)

The 19 strains of *S. suis* were subjected to Multilocus Sequence Typing (MLST), revealing 10 new allelic variants across seven housekeeping genes. The identified variants included one for the *aroA* gene, one for the *cpn60* gene, two for the *dpr* gene, three for the *gki* gene, one for the *mutS* gene, and two for the *recA* gene. These newly identified sequences for these housekeeping genes have been submitted to PubMLST for validation. Once verified, the allelic configurations of these new sequences will be registered in PubMLST, defining the nine new sequence types (STs) discovered in this study.

Results from the analysis of 19 *S. suis* isolates indicated that *aroA*, *cpn60*, *dpr*, *gki*, *mutS*, *recA*, and *thrA* genes possess 13, 14, 14, 14, 12, 13, and 12 distinct allelic variants, respectively, resulting in 17 different STs. Out of the 19 isolates, two strains were identified as ST28 and ST243, while the remaining ST types (ST2346, ST621, ST117, ST308, ST2339, ST477, ST2344, ST2345, ST2236, ST2347, ST353, ST2349, ST2350, ST2353, and ST2354) each had one strain. ST2346, ST2339, ST2344, ST2345, ST2347, ST2349, ST2350, ST2353, and ST2354 are newly identified STs in this study.

### 3.4. Pan-Genome and SNP Analysis

**Figure 3:**
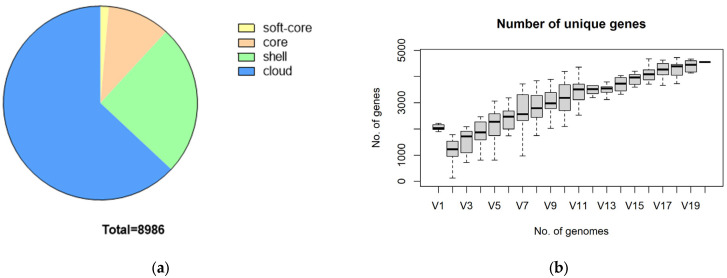

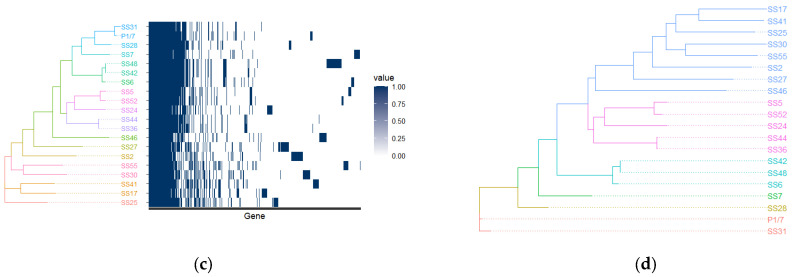
*S. suis* pan-genome. (**a**) The number of genes belonging to the core, the soft core, the shell, and the cloud of the *S. suis* species is pictured as a pie chart; (**b**) The relationship between the number of unique genes and the number of genomes; (**c**) The construction of the phylogenetic tree of the core genes of *S. suis*; Note: The tree on the left is a neighbor-joining phylogenetic tree constructed based on the core genes. The matrix plot on the right shows the presence and absence of genes on each strain in blue and white; (**d**) The SNP phylogenetic tree of 20 *S. suis* strains.

Pan-genome analysis of *S. suis* revealed the following gene distributions: The *S. suis* pangenome contains a total of 8986 genes, consisting of 939 core genes, 125 soft genes, 2259 shell genes, and 5663 cloud genes. The substantial number of cloud genes indicates significant heterogeneity among the 19 *S. suis* strains studied, underscoring the pan-genome’s “open” nature (Figure 3a). Increased strains lead to a rise in unique genes, reflecting the genomic diversity among the 19 isolated bacterial strains (Figure 3b). Utilizing the FastTree RAxML model and ggtree for visual analysis, we constructed a phylogenetic tree of the core genes from 20 *S. suis* strains identified by ROARY. Notably, strain SS31 from Xiangyang, Hubei, shares considerable genetic similarity with the reference strain P1/7, indicating the closest genetic relationship based on core genes. Conversely, SS25 and P1/7 exhibit the lowest similarity, suggesting the most distant genetic relationship (Figure 3c). The core genetic phylogenetic tree produced by Roary exhibited remarkable consistency with the SNP results (Figure 3d) generated by Parsnp software v1.5.3 [19], indicating that the majority of genomic variation may exist in the form of SNPs.

### 3.5. Antimicrobial Susceptibility Testing

**Figure 4:**
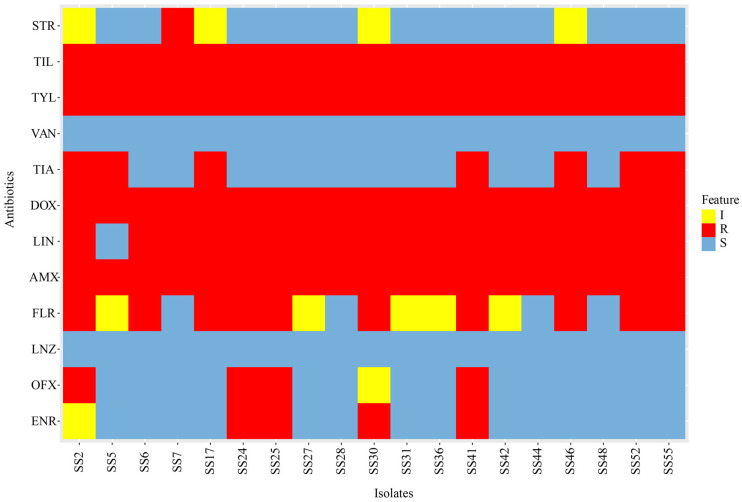
Heat map showing antimicrobial susceptibility profiles of *S. suis* strains. Rows represent bacterial strains, and columns represent antibiotics, where blue blocks indicate antibiotic susceptibility (S), yellow blocks indicate intermediacy (I), and red blocks indicate resistance (R) to action of antibiotics. ENR = Enrofloxacin; OFX = Ofloxacin; LNZ = Linezolid; FLR = Florfenicol; AMX = Amoxicillin; LIN = Lincomycin; DOX = Doxycycline; TIA = Tiamulin; VAN = Vancomycin; TYL = Tylosin; TIL = Tilmicosin; STR = Streptomycin.

The antimicrobial susceptibility of 19 isolates of *S. suis* was determined using the broth microdilution method according to CLSI standards, with the use of 12 antibiotic drugs. The results of antibiotic sensitivity for each drug are shown in Figure 4. The specific MIC value of each strain is listed in Supplemental Table S2. More than 70% of the isolates exhibited sensitivity to VAN (100.00%), LNZ (100.00%), ENR (73.68%), and STR (73.68%). These data provide insights into the treatment of *S. suis* infections. Additionally, a high frequency of resistance was observed for some antibiotics, with AMX, DOX, TLY, and TIL showing the highest resistance rates at 100.00%. The intermediate sensitivity level to FLR was the highest at 26.32% (5/19). All isolates of *S. suis* exhibited resistance to at least one antibiotic. However, none of the isolates showed resistance to all 12 tested antibiotics. As shown in Table 1, all 19 isolates displayed multidrug resistance (strains show resistance to, greater than, or equal to three classes of antibiotics). Among these isolates, eight strains (42.11%) exhibited resistance to four classes of antibiotics, which was the highest proportion, followed by seven strains (36.84%) of *S. suis* showing resistance to six classes of antibiotics. Notably, 2 isolates among the 19 exhibited multidrug resistance to antibiotics from seven different classes. Distribution of resistance types is summarized in Table 1. The results revealed nine different antibiotic resistance patterns. Among these, the resistance pattern (TYL-TIL)-LIN-AMX-DOX, observed in seven isolates (36.84%), was the most common resistance pattern observed among the isolates.

### 3.6. Detection of Drug Resistance Genes

To identify resistance genes, we analyzed the genomes of 19 isolates of *S. suis* using the online tool ResFinder. A total of 19 resistance genes were detected, including *tet*(O/W/32/O), *tet*(40), *tet*(O), *tet*(32), *erm*(A), *erm*(B), *mef*(A), *msr*(D), *aac*(6′)-*aph*(2″), *aph*(3′)-III, *ant*(6)-Ia, *cat*(pC221), *cat*, *cat*(pC194), *lnu*(B), *lsa*(E), and *optrA*. All these genes have been reported in resistant strains of *S. suis* [2,12,29]. The types of carried resistance genes in *S. suis* include seven classes (Table 2), namely Tetracycline, Aminoglycoside, Macrolide/lincosamide, Phenicol, Oxazolidinone, and Pleuromutilines. The major types of resistance genes are Tetracycline, Macrolide/lincosamide, and Aminoglycoside. All strains carried Tetracycline resistance genes (100%, 19/19), with the most prevalent resistance gene being *tet*(O) (89.47%, 17/19), followed by Macrolide/lincosamide (94.74%, 18/19) and Aminoglycoside (94.74%, 18/19) resistance genes, with the most prevalent resistance gene being *erm*(B) (94.74%, 18/19). Aminoglycoside (47.37%, 9/19), Phenicol (36.84%, 7/19), Oxazolidinone (26.32%, 5/19), and Pleuromutilines (15.79%, 3/19) also constituted a specific proportion as indicated in Table 2.

### 3.7. Identification of Core Genes of ICEs

**Figure 5:**
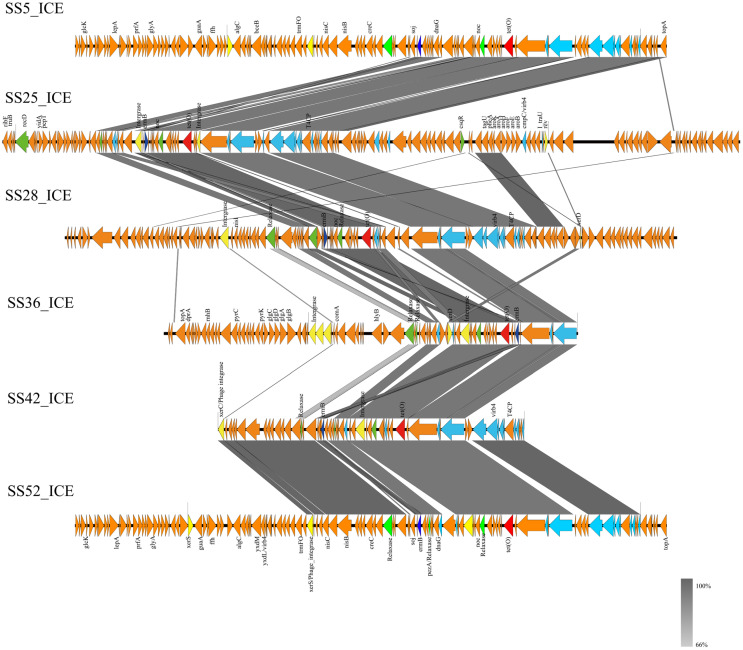
Comparison of ICEs carrying AMR genes. Light blue arrows indicate genes coding for Type IV Secretion System; green arrows indicate genes coding for relaxases; yellow arrows indicate genes coding for integrases; red arrows indicate *tet*(O) genes; and dark blue arrows indicate *erm*(B) genes.

ICEberg was used to predict and analyze the Integrative and Conjugative Elements (ICEs) in terms of their composition modules (Integration and Excision modules, Conjugative Pair formation modules, DNA processing and movement modules, and Regulatory modules). Table S3 demonstrates that among all the *S. suis* strains, fourteen carry ICE, while two strains only carry IME, resulting in a total carriage rate of 73.68%. Additionally, two strains carry incomplete ICE with a carriage rate of 10.53%. Strains not carrying ICE or IME account for only 15.79% (3/19). The overall carriage rate of ICE and IME in clinical isolates reaches 84.21% [(14/19) + (2/19)]. Table S4 shows that eight strains of *S. suis* carrying ICE or IME harbor at least one or more antimicrobial resistance genes, such as *ant*6, *aph*3, *cat*, *erm*(B), and *tet*(O). Notably, six ICEs carry both *erm*(B) and *tet*(O). Although ICEs exhibit different gene content and length, they share regions coding for the Type IV Secretion System (Figure 5).

## 4. Discussion

*S. suis* is a significant zoonotic pathogen with a global impact, affecting both humans and pigs. It can be carried by pigs regardless of their health status, whether diseased, healthy, or clinically recovered. It primarily causes meningitis, septicemia, polyserositis, arthritis, and endocarditis in weaned piglets, inflicting considerable economic losses on the swine industry [30]. The overuse of antibiotics exacerbates issues of bacterial resistance and complicates the management of horizontal gene transfer of resistance genes [12,31]. Asymptomatic pigs play a crucial role as a source of infection, heightening the risk of potential outbreaks or transmission to individuals near pigs or pork products [32,33]. Moreover, asymptomatic pigs can harbor multidrug-resistant strains of *S. suis*, presenting challenges to its control. Therefore, continuous monitoring of *S. suis* resistance (AMR) and research into resistance mechanisms is imperative [34,35]. 

Hubei, a prominent pig production and transportation center in China [36], recorded a total annual pig production of 42.86 million in 2022. However, little well-directed data regarding the genomics and AMR of *S. suis* have been reported in Hubei Province. Therefore, monitoring Hubei province *S. suis* is crucial to ensuring the healthy development of pig farming in China. 

In order to investigate the genetic characteristics and epidemiology of *S. suis*, we collected 100 tissue samples from diseased animals across various farms in Hubei. The *rec*N gene and the 16S rRNA gene were employed for the identification of *S. suis* strains. A total of 19 *S. suis* strains were identified through PCR amplification and sequencing. The utilization of the *rec*N gene was based on its low similarity at the species level and high differentiation at the subspecies level, facilitating the discrimination between *S. suis* and non-*S. suis* strains [37]. Furthermore, the 336 bp size of *rec*N PCR products allows for reduced PCR reaction times. The 16S rRNA gene, a highly conserved sequence within bacterial genomes, is widespread among prokaryotes and carries crucial phylogenetic information. While its sequence demonstrates significant conservation across various bacterial species, it also exhibits adequate variation at the species or genus level, making it an ideal marker for bacterial differentiation. Although 16S rRNA sequencing is commonly employed in bacterial identification and classification, it is limited by its inability to provide sufficient resolution in distinguishing certain species or subspecies [14]. Therefore, we combined *rec*N-specific primers with 16S rRNA to enhance the accuracy of microbial identification [38]. Advancements in sequencing technology have substantially decreased the costs associated with whole-genome sequencing (WGS), broadening its utility across various research domain [39]. WGS plays a crucial role in tracing pathogen transmission and evolution, identifying sources of outbreaks, and monitoring the emergence of drug resistance. Furthermore, it serves as a fundamental tool in genomics, evolutionary biology, and conservation genetics, enabling the identification of novel genes, elucidation of genetic diversity, and exploration of evolutionary relationships [40].

Pan-genome analysis, elucidating core and unique bacterial traits, offers a holistic comprehension of pathogen diversity and evolution. This comprehensive understanding aids in refining vaccine development and optimizing antibiotic strategies [41]. Roary was employed for pan-genome analysis of the P1/7 strain and 19 isolates of *S. suis* from Hubei. In this study, Roary was utilized for pan-genome analysis of the P1/7 strain and 19 isolates of *S. suis* from Hubei. The pan-genome analysis revealed 5663 cloud genes among the studied strains, representing approximately 63% of the total genes, indicating an open genome characteristic of *S. suis*, consistent with previous research conducted by Guo [9]. Additionally, the SNP analysis results of 20 strains of *S. suis* confirmed our pan-genome study results and enhanced the reliability of our analyses.

The serological properties of bacterial capsular polysaccharides are crucial for serotyping *S. suis*. Currently, 29 serotypes of *S. suis* have been identified. Among these, serotype 2 is notably associated with infections in both pigs and humans [42]. Serotype 9 has become an essential and prevalent serotype causing disease in pigs in many European countries [32,43]. An increase in the prevalence of serotype 9 isolates from diseased pigs has been observed in the Netherlands, Spain, and Germany [32]. It is also a commonly found serotype in clinically diseased pigs in Chinese commercial swine farms [44]. However, a human case due to *S. suis* serotype 9 was recently reported in Thailand [45]. In this study, a total of 10 different serotypes were identified, with serotype 9 being the predominant one, accounting for 21.05% of the cases.

Through MLST analysis of 19 isolated strains of *S. suis*, we have identified nine new MLST (ST2346, ST2339, ST2344, ST2345, ST2347, ST2349, ST2350, ST2353, and ST2354), thereby substantially enriching the MLST dataset of *S. suis*. Importantly, these isolated strains were obtained from diseased animals, and whether the newly identified MLST of *S. suis* strains are associated with the development of animal diseases remains unclear and requires further investigation. The combined analysis of drug susceptibility tests and resistance genes suggests that the spectrum of resistance genes in clinical isolates is not always directly associated with specific phenotypes, indicating the presence of additional resistance mechanisms in *S. suis* [46]. The antimicrobial resistance gene types carried by Integrative and Conjugative Elements (ICEs) and Integrative and Mobilizable Elements (IMEs) of the 19 *S. suis* isolates were analyzed using ResFinder. Notably, the ICE elements of six *S. suis* strains carry both *erm*(B) and *tet*(O), potentially making a significant contribution to the high resistance of *S. suis* to macrolides and tetracyclines.

In this study, we identified novel STs of *S. suis*, potentially linked to the progression of related diseases. Utilizing whole-genome sequencing and pan-genome analyses, we deepened our understanding of the pan-genomic characteristics of *S. suis*. Our investigation of Integrative and Conjugative Elements (ICEs) mobile elements has provided valuable insights into understanding the dissemination of resistance genes. This study delves into the epidemiology, antimicrobial resistance patterns, and population dynamics, including serotypes, sequence types, and genomic characteristics, of *S. suis* within Hubei Province, China, at a population scale. The primary objective of this research is to enrich the genetic database of *S. suis*, deepen our understanding of its population structure and genetic evolutionary traits, and provide essential groundwork and empirical support for the comprehensive prevention and management of *S. suis* infections in China.

## Figures and Tables

**Table 1:** Drug resistance profiles of *S. suis* strains isolated from clinically healthy pigs.

Serial Number	No. of Antimicrobials	No. of Strains	Antimicrobial Resistotypes *
1	4	1	(TYL-TIL)-TIA-AMX-DOX
2	4	7	(TYL-TIL)-LIN-AMX-DOX
3	5	1	(TYL-TIL)-LIN-AMX-FLR-DOX
4	5	1	(TYL-TIL)-LIN-AMX-STR-DOX
5	6	4	(TYL-TIL)-LIN-TIA-AMX-FLR-DOX
6	6	1	(TYL-TIL)-LIN-AMX-ENR-FLR-DOX
7	6	2	(TYL-TIL)-LIN-AMX-(ENR-OFX)-FLR-DOX
8	7	1	(TYL-TIL)-LIN-TIA-AMX-OFX-FLR-DOX
9	7	1	(TYL-TIL)-LIN-TIA-AMX-(ENR-OFX)-FLR-DOX

* TYL = Tylosin; TIL = Tilmicosin; LIN = Lincomycin; TIA = Tiamulin; AMX = Amoxicillin; ENR = Enrofloxacin; OFX = Ofloxacin; FLR = Florfenicol; STR = Streptomycin; DOX = Doxycycline. The antibiotics within parentheses belong to the same class.

**Table 2:** AMR genes detected in 19 isolates of *S. suis* recovered in Hubei and their association with antibiotic resistance.

AMR Genes	Antibiotic Family	Antibiotic Tested	+/R *	+/S *	+/I *
*tet*(32)	Tetracycline	Doxycycline	19/19	/	/
*tet*(O/W/32/O)					
*tet*(40)					
*tet*(O)					
*erm*(A)	Macrolides	Tylosin; Tilmicosin	18/19	/	/
*erm*(B)					
*mef*(A)					
*msr*(D)					
*erm*(A)	Lincosamides	Lincomycin	17/18	1/1	/
*erm*(B)					
*lnu*(B)					
*lsa*(E)					
*lsa*(E)	Pleuromutilines	Tiamulin	2/7	1/12	/
*aac*(6′)-*aph*(2″)	Aminoglycoside	Streptomycin	0/1	5/14	4/4
*aph*(3′)-III					
*ant*(6)-Ia					
*cat*(pC221)	Phenicol	Florfenicol	4/10	0/4	2/5
*cat*					
*cat*(pC194)					
*optrA*					
*optrA*	Oxazolidinone	Linezolid	/	5/19	/

* The population was distributed in resistant (R), susceptible (S), or intermediate (I) isolates for each antibiotic. The number of isolates with particular AMR genes (“+”) in each population is indicated.

## Data Availability

The original contributions presented in this study are included in this article/Supplementary Material; further inquiries can be directed to the corresponding author/s.

## Supplementary Materials

The following supporting information can be downloaded at: https://www.mdpi.com/article/10.3390/microorganisms12050917/s1, Table S1: The information of 19 *S. suis* isolates; Table S2: Minimum inhibitory concentrations (MIC) of tested antimicrobial agents for the studied bacterial; Table S3: ICE/IME detected in 19 isolates of *S. suis* recovered in Hubei; Table S4: AMR genes detected in ICE/IME of *S. suis* recovered in Hubei.
