# Supplementary material for: Isolation, Identification, Genomic Diversity, and Antimicrobial Resistance Analysis of Streptococcus suis in Hubei Province of China from 2021 to 2023
Source: Microorganisms. 2024 Apr 30;12(5):917. doi: 10.3390/microorganisms12050917 (PMC11124115; doi:10.3390/microorganisms12050917)
Supplement: Supplementary file 1 [file microorganisms-12-00917-s001.zip › Table S1; Table S3; Table S4.pdf]

Table S1 The information of 19 *S. suis* isolates.

Isolate	Year	Source	Location
SS2	2021	lung+lymph nodes+spleen	Huanggang
SS5	2021	joint fluid+pericardial effusion	Huangshi
SS6	2021	lung	Xianning
SS7	2021	lung	Huanggang
SS17	2021	lung	Enshi
SS24	2022	lung	Xiaogan
SS25	2022	lung	Huangshi
SS27	2022	lung	Tianmen
SS28	2022	lung	Suizhou
SS30	2022	lung+pericardial effusion	Enshi
SS31	2022	lung	Xiangyang
SS36	2022	lung	Xiangyang
SS41	2022	lung	Huangshi
SS42	2022	lung	Xiaogan
SS44	2022	lung	Huangshi
SS46	2022	lung+pericardial effusion	Xianning
SS48	2022	lung	Suizhou
SS52	2022	lung	Huanggang
SS55	2023	lung	Wuhan

Table S3 ICE/IME detected in 19 isolates of *S. suis* recovered in Hubei.

Isolate	Length/bp	Tpye
SS2	182624	Putative ICE with T4SS
	57957	Putative ICE with T4SS
	29289	Putative ICE with T4SS
SS5	123195	Putative ICE with T4SS
	9681	Putative IME
SS6	78164	Putative ICE without identified DR
SS7	44803	Putative ICE with T4SS
	88819	Putative ICE with T4SS
SS24	99622	Putative ICE with T4SS
	115655	Putative ICE with T4SS
	84929	Putative ICE with T4SS
	45811	Putative ICE with T4SS
	13835	Putative IME without identified DR
	5669	Putative AICE with Rep and Tra
	3605	Putative IME without identified DR
	14929	Putative IME
SS25	153559	Putative ICE with T4SS
	9262	Putative IME
SS27	4917	Putative IME without identified DR
SS28	127406	Putative ICE with T4SS
	72030	Putative ICE with T4SS
SS30	90035	Putative ICE with T4SS
	83224	Putative ICE with T4SS
	93027	Putative ICE with T4SS
	27587	Putative IME
SS36	46744	Putative ICE with T4SS
	86205	Putative ICE with T4SS
SS41	3893	Putative IME without identified DR
SS42	63662	Putative ICE without identified DR
	6115	Putative IME
	10875	Putative IME without identified DR
SS44	46744	Putative ICE with T4SS
SS48	66663	Putative ICE without identified DR
	6115	Putative IME
	10875	Putative IME without identified DR
SS52	123202	Putative ICE with T4SS
	89618	Putative ICE with T4SS
	8735	Putative IME
	11399	Putative IME without identified DR
	9681	Putative IME
SS55	38331	Putative IME without identified DR
	48259	Putative ICE with T4SS

Table S4 AMR genes detected in ICE/IME of *S. suis* recovered in Hubei.

Isolate	Tpye	AMR genes
SS5	Putative ICE with T4SS	ermB tet(O)
SS24	Putative IME	ANT6 APH3 CAT
SS25	Putative ICE with T4SS	ermB tet(O)
SS28	Putative ICE with T4SS	ermB tet(O)
SS30	Putative IME	ermB
SS36	Putative ICE with T4SS	ermB tet(O)
SS42	Putative ICE without identified DR	ermB tet(O)
SS52	Putative ICE with T4SS	ermB tet(O)
